# Cryo-EM of soft-landed β-galactosidase: Gas-phase and native structures are remarkably similar

**DOI:** 10.1126/sciadv.adl4628

**Published:** 2024-02-14

**Authors:** Tim K. Esser, Jan Böhning, Alpcan Önür, Dinesh K. Chinthapalli, Lukas Eriksson, Marko Grabarics, Paul Fremdling, Albert Konijnenberg, Alexander Makarov, Aurelien Botman, Christine Peter, Justin L. P. Benesch, Carol V. Robinson, Joseph Gault, Lindsay Baker, Tanmay A. M. Bharat, Stephan Rauschenbach

**Affiliations:** ^1^ Department of Chemistry, University of Oxford, South Parks Road, Oxford OX1 3QU, UK.; ^2^ Kavli Institute for NanoScience Discovery, Dorothy Crowfoot Hodgkin Building, Oxford OX1 3QU, UK.; ^3^ Thermo Fisher Scientific, 1 Boundary Park, Hemel Hempstead, Hertfordshire HP2 7GE, UK.; ^4^ Structural Studies Division, MRC Laboratory of Molecular Biology, Francis Crick Avenue, Cambridge CB2 0QH, UK.; ^5^ Department of Chemistry, University of Konstanz, Konstanz 78457, Germany.; ^6^ Thermo Fisher Scientific, De Schakel 2, 5651GH Eindhoven, Netherlands.; ^7^ Thermo Fisher Scientific, Bremen 28199, Germany.; ^8^ Biomolecular Mass Spectrometry and Proteomics, Bijvoet Centre for Biomolecular Research and Utrecht Institute for Pharmaceutical Sciences, Utrecht University, Padualaan 8, 3584 CH Utrecht, Netherlands.; ^9^ Thermo Fisher Scientific, 5350 NE Dawson Creek Drive, Hillsboro, OR 97124, USA.; ^10^ Department of Biochemistry, University of Oxford, Oxford OX1 3QU, UK.

## Abstract

Native mass spectrometry (MS) has become widely accepted in structural biology, providing information on stoichiometry, interactions, homogeneity, and shape of protein complexes. Yet, the fundamental assumption that proteins inside the mass spectrometer retain a structure faithful to native proteins in solution remains a matter of intense debate. Here, we reveal the gas-phase structure of β-galactosidase using single-particle cryo–electron microscopy (cryo-EM) down to 2.6-Å resolution, enabled by soft landing of mass-selected protein complexes onto cold transmission electron microscopy (TEM) grids followed by in situ ice coating. We find that large parts of the secondary and tertiary structure are retained from the solution. Dehydration-driven subunit reorientation leads to consistent compaction in the gas phase. By providing a direct link between high-resolution imaging and the capability to handle and select protein complexes that behave problematically in conventional sample preparation, the approach has the potential to expand the scope of both native mass spectrometry and cryo-EM.

## Teaser

Electrospray ion-beam deposition on cold grids and in-vacuum ice growth enable cryo-EM of mass-selected proteins at 2.6 Å.

## INTRODUCTION

Native mass spectrometry (MS) is based on native electrospray ionization (ESI), in which intact proteins and protein complexes are transferred gently from solution into the gas phase by the application of low electrospray potential, using low flow rates, aqueous solutions, volatile buffers, and, where appropriate, addition of membrane mimetics (*1*, *2*). Because native MS aims to provide information relevant to proteins in their native, solvated environment, results are often interpreted in the context of x-ray crystallography or cryo–electron microscopy (cryo-EM) structures (*3*–*7*). However, since the early days of native MS, the extent to which the native protein fold and interactions are preserved upon transfer into the gas phase has been a matter of intense investigation and debate (*8*–*13*).

Native MS comprises a range of analytical techniques that provide molecular structural information. Ion mobility spectrometry can demonstrate retention of the globular protein shape but also gas-phase collapse and unfolding can be observed, depending on charge state, degree of hydration, and temperature (*14*–*16*). Hydrogen-deuterium exchange reveals that similar amides are exposed or protected in solution and in the gas phase (*17*, *18*). Various forms of ion activation show retention of native stoichiometry and conformation (*19*). In addition, molecular dynamics (MD) studies suggest that proteins adopt kinetically trapped, compacted gas-phase structures that preserve the native secondary structure to a large extent (*13*, *20**–**25*). In particular, formation of additional intramolecular hydrogen bonds, also known as self-solvation, following dehydration has been proposed (*26*).

Together, these findings suggest that near-native structures can be retained in the gas phase. However, because of the lack of spatial resolution in experimental MS data, the structural detail in MD simulations is difficult to validate at the atomic level and thus the degree to which biologically relevant structural motifs are retained in gas-phase protein ions remains unclear. Furthermore, this limitation has prevented access to a detailed molecular picture of the role of solvation in protein structure and folding.

**Fig. 1.**
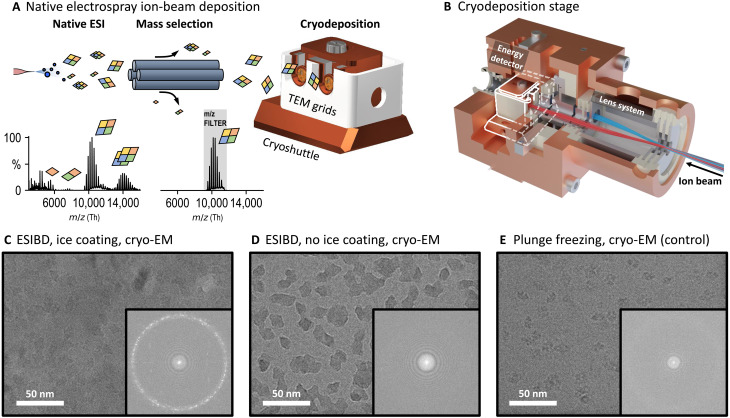
Determination of solution and gas-phase protein structure. (**A**) Native ESIBD workflow. Ions are transferred into the gas phase using native electrospray, mass-selected, and deposited with controlled energy, density, and distribution on cold grids inside the cryoshuttle. Full and mass-selected spectra of β-galactosidase are shown. (**B**) Render of cryogenically cooled landing stage. Protein ion beam is guided to the two grid positions using an electrostatic lens system. The stage is cooling grids and shielding them from contamination and radiation. The position of the cryoshuttle inside the cryostage is highlighted in white. (**C**) Cryo-EM micrograph from native ESIBD sample with ice growth and corresponding power spectrum, showing nonvitreous ice due to temperature increase during transfer. (**D**) Cryo-EM micrograph from native ESIBD sample without ice growth and corresponding power spectrum, showing minimal ice. (**E**) Cryo-EM micrograph obtained from plunge-frozen sample and corresponding power spectrum showing vitreous ice.

To enable a detailed comparison of native solution structure and near-native gas-phase structure, soft-landing native electrospray ion-beam deposition (ESIBD) of protein ions directly onto cryo-EM grids has been suggested (*27*). In native ESIBD, ions produced by native ESI are mass-to-charge (*m/z*)–filtered and thermalized in a collision cell to obtain a molecular ion beam of narrow energy distribution (Fig. 1A). The ion beam is then deposited with a very low deposition energy onto a substrate in vacuum. This approach has the potential to establish a direct link between chemical information from native MS and structural information from cryo-EM.

Already, direct combination of native MS and various analysis methods revealed retention of globular shape and biological activity of proteins and viruses (*28**–**31*). More recently, improved workflows using cryo-EM, negative-stain transmission electron microscopy (TEM), or low-energy electron holography have demonstrated that protein complexes retain their overall shape at a resolution in the nanometer range (*31**–**36*). However, in the absence of a method that provides structures of proteins at the level of side-chain resolution, the extent to which the solution structure is retained in the native ESIBD process has remained unclear.

Here, we present a 2.6-Å resolution cryo-EM density map of the tetrameric protein complex β-galactosidase, obtained from a sample prepared by in-vacuum deposition of a mass-selected, gas-phase protein ion beam onto a cryo-EM grid. This result is enabled by our native ESIBD instrumentation, which delivers proteins at very low translational energy onto substrates held at cryogenic temperatures, then coats them with ice grown from water vapour, and finally transfers them through a controlled inert atmosphere into liquid nitrogen. The three-dimensional (3D) map obtained from our data shows the structure of the adsorbed, gas-phase protein ion, which is remarkably similar to the structure obtained when using conventional, plunge-freezing sample preparation. The local fold and the integrity of the subunits are retained. Differences are observed at the protein surface and in the relative arrangement of the subunits. MD simulations suggest that these changes correlate to compaction of previously water-filled cavities and grooves upon dehydration. Remarkably, the resolution obtained after averaging more than 400,000 single-particle images of β-galactosidase suggest the formation of a single, well-defined, gas-phase structure under the conditions described herein.

## RESULTS

### ESIBD workflow

We performed native ESIBD using a previously described, modified, commercial mass spectrometer (Thermo Scientific Q Exactive UHMRinstrument), which generates intense, *m/z*-filtered, and thermalized ion beams (*32*, *37*). This instrument was extended by a cryogenically cooled deposition stage, shown in Fig. 1B (complete system shown in fig. S1). TEM grids with 2-nm amorphous carbon films suspended on a holey film are held at cryogenic temperatures (*T* = 130 K) in ultrahigh vacuum (*P* <10^−9^ mbar). Compared to deposition at room temperature, cryogenic conditions suppress thermal diffusion and structural fluctuations immediately after deposition and during sample transfer (*38*).

Up to two TEM grids were loaded and retrieved using a custom cryoshuttle, compatible with Thermo Scientific Aquilos hardware (*39*). Deposition began after optimizing *m/z* selection, ion-beam intensity, and deposition energy (*32*). In contrast to the conventional plunge-freezing procedure, where grid, solvent, and proteins are frozen simultaneously, protein ions freeze individually as they land on the already cold substrate. After deposition, the shuttle passed from the vacuum of the deposition chamber through clean nitrogen gas into liquid nitrogen, without exposure to ambient conditions. During sample transfer, grids were shielded from heat and contaminants using a mechanical shutter mechanism within the shuttle (fig. S2). To coat the cold samples with thin layers of ice, the partial pressure of water was temporarily increased while keeping the shuttle inside the stage.

### Structure determination

Using our native ESIBD workflow, we prepared samples of β-galactosidase with and without coating in ice (see Fig. 1, C and D). A third sample was prepared using the conventional plunge-freezing workflow, starting with the same protein solution (see Fig. 1E). The data were recorded on a Krios 300 kV cryo-TEM (Thermo Scientific) and processed using cryoSPARC (see Materials and Methods) (*40*).

All samples show similar particle shapes, density, and distribution. The ice-free native ESIBD sample provides substantially higher contrast than those with ice. The absence of ice, and thus cleanliness of the sample transfer, is also confirmed by the absence of ice signals in the power spectrum. However, data from this sample did not result in high resolution, showing the molecular envelope with only rudimentary internal features and surrounded with a high-density shell, likely caused by the presence of a few monolayers of ice captured in vacuum (see figs. S5 and S6). Thus, we will focus on the comparison of the ice-coated, native ESIBD and plunge-frozen control samples in the following.

**Fig. 2.**
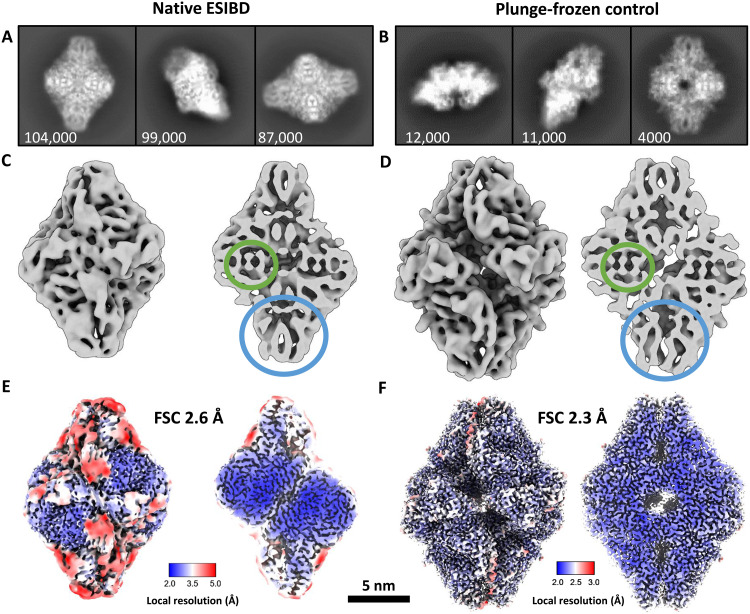
Comparison of 2D classes and 3D maps from native ESIBD and plunge-frozen control samples. (**A** and **B**) Selected 2D classes (number of particles indicated). (**C** and **D**) Cryo-EM density maps (low pass–filtered to 8 Å to improve visibility of features) corresponding to the 2D classes above. α Helices and β sheets are highlighted by green and blue circles, respectively. The native ESIBD sample shows overall smaller size and reduced dimensions of grooves and the central cavity. (**E** and **F**) High-resolution density maps (2.6- and 2.3-Å resolution estimated by gold-standard FSC). Color shows local resolution. Less resolved regions in the native ESIBD sample (E) coincide with typically solvated areas at the protein surface. Decreased resolution in these regions, though less pronounced, can also be seen for the plunge-frozen sample (F). All maps are scaled consistently as indicated by the shared scale bar. Movie S1 shows an animation of the 2.6-Å map.

The corresponding micrographs in Fig. 1 (C and E) show similar contrast. The presence of nonvitreous ice in the native ESIBD sample, revealed by diffraction spots in the power spectrum, indicates an increase of grid temperature to more than −140°C during sample transfer. Corresponding 2D classes are shown in Fig. 2 (A and B). The 2D classes of the native ESIBD sample show similar preferred orientation but substantially more structural detail compared to previous studies where deposition and transfer were performed at room temperature (*33*). Clearly defined elements of the secondary structure are identified in the 2D classes of both samples. In particular, the diamond-shaped classes show features that directly correlate to the central α helices and β sheets at the tips. Reductions of overall size of the complex and the central cavity are the main differences observed in 2D between native ESIBD and plunge-frozen sample.

3D cryo-EM density maps (Fig. 2, C to F) for the native ESIBD and the plunge-frozen control sample were obtained with a resolution of 2.6 and 2.3 Å, respectively. The 3D map for the control sample is in very good agreement with the previously published model [Protein Data Bank (PDB) 6CVM] (*41*). Both maps clearly show secondary structure, including α helices and β sheets (highlighted in Fig. 2, C and D). Native ESIBD led to a compaction of about 10, 14, and 20% along the long, short, and intermediate principal axis, respectively. The size of the central cavity is decreased by 40%. Local resolution plots (Fig. 2, E and F) indicate that the core regions of the subunits are highly ordered and resolved at below 3 Å, showing information on the side-chain level. However, surface-exposed areas that typically interact with solvent have poorer local resolution, suggesting local structural heterogeneity.

### Solution versus gas-phase structures

**Fig. 3.**
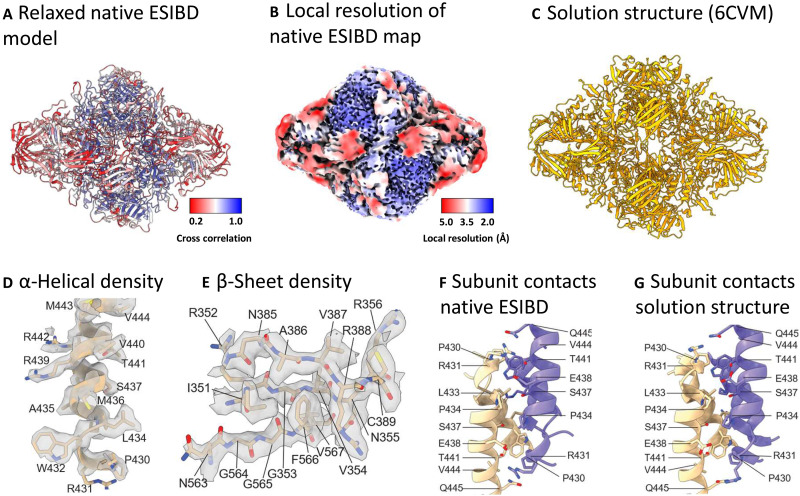
Atomic model of β-galactosidase from native ESIBD. (**A**) Relaxed full-length atomic model, created by MD flexible fitting of the solution structure into the 2.6-Å native ESIBD map using ISOLDE. Color indicates goodness of fit of the relaxed model into the native ESIBD density, quantified by refinement of the atomic model against the density in Phenix and plotting the resulting per-residue cross-correlation value onto the model. Better fits for the model can be seen in the inner core region of the complex. (**B**) Color-coded local resolution map from native ESIBD, indicating strong correlation between resolution and quality of the relaxed model. (**C**) Solution structure (PDB 6CVM). (**D** and **E**) α-Helical and β sheet segment from a manually built atomic model within the density at 16 σ contour level. (**F** and **G**) Contacts between two helix pairs at the interface between two subunits in the built atomic versus the solution model. Side chains are shown for residues extending toward the interface.

Next, we investigated structural differences between the solution structure and the native ESIBD structure in more detail. By relaxing the β-galactosidase PDB model 6CVM (Fig. 3C) into the map from native ESIBD using MD flexible fitting in ISOLDE (*42*), we generated a tentative model, shown in Fig. 3A. It is more compact compared to the solution model, but the original secondary structure features are preserved and fit the core of the map well as seen by the color-coded cross-correlation. Movie S2 shows an animation of the original and relaxed model in the corresponding maps (low pass–filtered to 8 Å) as well as an extrapolated transition between them, visualizing the structural change during native ESIBD.

While the surface-exposed areas of the map did not show sufficient side-chain densities to enable model building, an unambiguous atomic model of the well-resolved core (300 residues) was built, with good agreement between map and model [model-versus-map Fourier shell correlation (FSC): 2.6 Å, see table S1 and fig. S7]. The resulting model indicates retention of secondary and tertiary structure motifs. In particular, α helices and β sheets (Fig. 3, D and E) and main-chain segments [root mean square deviation (RMSD) below 1 Å; fig. S8, A and C] in the well-ordered monomer core show excellent local agreement between ESIBD and solution structure. Several contacts between subunits in the tetramer are retained from the solution structure (Fig. 3, F and G). While one of the two subunit interfaces of each subunit appears to be similar to the solution structure (RMSD: 2.8 Å), the second one deviates more substantially (RMSD: 6.6 Å), but consistently (see fig. S8B). Together, these data suggest that the core of each subunit stays intact and highly ordered, while surface-exposed parts of the protein show reduced order. Overall, the architecture of the tetramer is maintained, but largely consistent variations in subunit interfaces cause a compaction of the complex, during which formerly water-filled cavities and grooves reduce in size.

### The effect of dehydration on structure

**Fig. 4.**
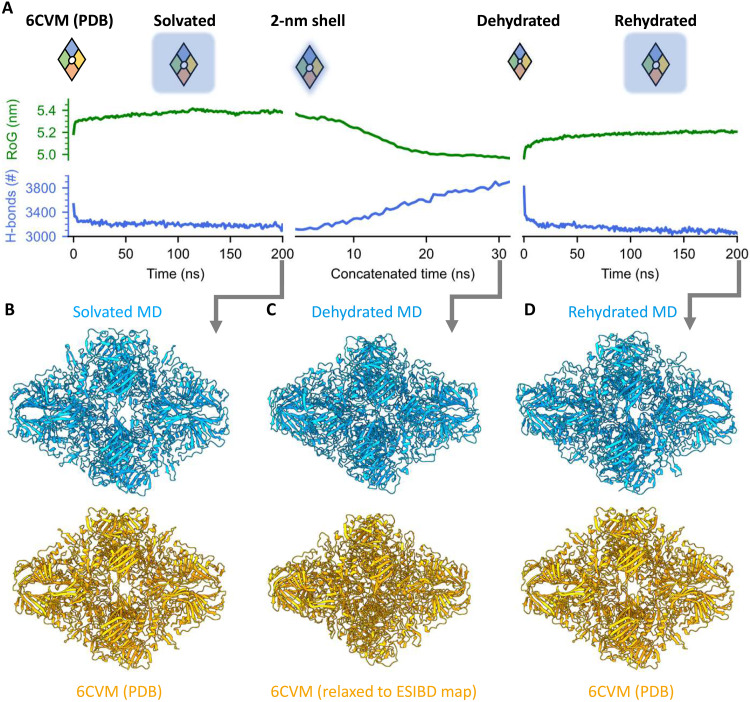
MD simulation of structural changes of β-galactosidase during solvation, dehydration, and rehydration. (**A**) Evolution of the RoG (green) and number of intramolecular hydrogen bonds (blue) in the three phases of the MD simulation. Hydration state and size of β-galactosidase are indicated schematically. (**B** to **D**) Ribbon diagrams for models from simulation and experiment are shown in blue (top) and orange (bottom), respectively. (B) After solvation of 6CVM for 200 ns, only minor structural changes are observed. (C) The solvated model is gradually dehydrated, resulting in a compacted model, very similar to the native ESIBD model obtained from flexible fitting. (D) The dehydrated model was then allowed to rehydrate over 200 ns, largely recovering the secondary structure of 6CVM. See movie S3 for a morph and table S1 for RMSD values between the models shown here.

To investigate the effect of changing solvation during the ESI process, we performed MD simulations of the dehydration and rehydration of β-galactosidase (see Materials and Methods for details). The simulations are based on the PDB model 6CVM (*41*), which represents the state of β-galactosidase captured in our map of the plunge-frozen control sample, and are independent from the experimental, native ESIBD cryo-EM density map. Figure 4 shows the evolution of the radius of gyration (RoG) and number of intramolecular hydrogen bonds and compares models from simulation and experiment.

First, we prepared a solvated initial model at 300 K (Fig. 4, A and B, left) after protonating 44 surface residues, corresponding to the dominating charge state observed in the experiment. Equilibration in solvent caused only minor changes to RoG, hydrogen bond number, and overall structure compared to 6CVM.

To generate a structural model of the gas-phase state of the protein, we dehydrated the initial structure at constant protonation state in short, free, atomistic simulations at 370 K following an adapted trajectory stitching approach (Fig. 4, A and C, middle) (*43*). Here, in 64 simulation windows of 500 ps each, water molecules beyond a 2-nm shell around the protein surface were gradually removed until the amount of residual water converged to about 1400 molecules that could not be evaporated from the protein without increasing the simulation temperature. At the end of the simulation, there is no more hydration shell and the remaining water molecules are located inside the protein structure (see fig. S9).

After dehydration, the outer dimensions and internal features—in particular grooves, central cavity, α helices, and β sheets—closely resemble the features of the native ESIBD cryo-EM density map. A decrease of the RoG by 7% and formation of 700 additional intramolecular hydrogen bonds indicate compaction and self-solvation, respectively.

Next, we simulated the rehydration of the dehydrated MD structure of β-galactosidase, motivated by previous work that has shown that rehydration of gas-phase structures, in silico or in experiments, can largely recover native structural motifs, collision cross sections, and biological activity (*10*, *20**–**23*, *37*, *44*, *45*). Rehydration over 200 ns (Fig. 4, A and D, right) led to restoration of the central cavity, outer dimensions, and recovery of many tertiary structure features of the native hydrated protein complex. The original number of intramolecular hydrogen bonds is recovered within the first 10 ns and keeps decreasing slightly afterward, while the decrease in RoG is partially reversed. Movie S3 shows a morph between the three states shown in Fig. 4, highlighting the similarity of changes in overall size, extent of cavities and grooves, and subunit orientation.

## DISCUSSION

Combining cryo-EM at side-chain resolution and molecular simulations allows us to understand how the structure of a protein is affected by the native ESIBD workflow. When water is removed, the protein compacts as a consequence of the relative motion of its subunits maximizing their mutual interaction. In this process, cavities and grooves that were previously filled with water are closed to form new intramolecular interfaces. Thus, structural changes are generally observed at the periphery of subunits where the molecule has been exposed to solvent before and is thus susceptible to self-solvation upon dehydration (*15*, *26*, *46*, *47*).

On the basis of the close match of experimental and MD structures, we conclude that the observed change is mostly driven by dehydration, as it is the only effect considered in our MD simulations. Landing and substrate interaction are key parameters of the native ESIBD workflow and might contribute to the lower resolution at the protein surface. However, for β-galactosidase under native ESIBD conditions, i.e., 2 eV per charge deposition energy, on a thin carbon support, and cryogenic temperature, landing and substrate interaction contribute to a substantially smaller extent than dehydration to the overall structural changes.

Because it is based on averaging, single-particle cryo-EM requires a chemically and structurally homogeneous set of single-particle images to achieve high resolution. Our experiment shows that native ESIBD can produce samples with sufficient homogeneity to derive atomic models using cryo-EM at 2.6-Å resolution. The dehydrated gas-phase state is similar to the solution structure and in particular retains the local fold and secondary structure features. Our cryo-EM structural data represent a direct, experimental validation of structural, native MS, which generally provides data at lower spatial resolution. Using rehydration in silico, we have shown that the native structure can largely be recovered from the dehydrated structure. Together with the partial atomic model, this demonstrates the possibility of determination of solvated protein structures from cryo-EM via native ESIBD, without the requirement of a priori knowledge beyond the protein sequence.

The high-resolution structural information obtained via native ESIBD will enable more detailed investigation of the effects of gas-phase activation, deposition energy, temperature, substrate, and postlanding modification steps. Further insights are to be expected from extending MD simulations to include landing and substrate interactions. Performance and information content of native ESIBD combined with cryo-EM may be improved by using higher ion transmission (*48*), ion mobility filtering (*49*), cross-linking (*50*), and low-temperature membrane substrates tailored for efficient deposition energy dissipation, weak surface interaction, and low surface diffusion rates (*38*, *51*, *52*). Preferred particle orientation, likely due to maximization of van der Waals interaction with the carbon substrate in vacuum, might be addressed in the future by using rough substrates, stage tilting, or particle reorientation during rehydration.

In conventional cryo-EM sample preparation, poor sample quality is often directly linked to the plunge-freezing workflow. Thinning and freezing the solvent film can result in inconsistent ice thickness, ice-related radiation-induced motion, and denaturation at the substrate-solvent and air-solvent interfaces (*53*–*55*). It is important to note that adding ice gradually from individual gas-phase water molecules instead does not expose particles to an air-solvent interface and particles retain their orientation and hydration state. It also prevents buildup of tension in the ice and provides the option to control the thickness of the ice layer. Capitalizing on established MS workflows, native ESIBD has the potential to circumvent plunge-freezing related limitations of grid quality and preparation reproducibility, and allow for much higher control of sample purity, particle number, and particle distribution. Thus, native ESIBD may enable determination of structures of proteins that are not amenable to the conventional plunge-freezing workflow.

Comparison of our samples with and without ice underpins the importance of controlled modification of native ESIBD samples by the addition of water-ice or another matrix after landing. Compared to plunge freezing, the growth of ice layers can be controlled with much greater precision in vacuum to obtain optimal phase and thickness by regulating partial pressure of water vapor, growth temperature, and time (*56*). In particular, lower temperatures and increased transfer speed will allow to maintain vitreous ice, which has been previously demonstrated for the Aquilos sample transfer system (*39*). Other variations of the workflow, including matrix landing, protein hydration in the gas phase, and controlled rehydration after landing, may allow for a complete experimental route to native, hydrated protein structures while retaining the advantages of gas-phase control.

## MATERIALS AND METHODS

### Protein preparation

β-Galactosidase (G3153-5MG) was purchased from Sigma-Aldrich. The lyophilized powder was resuspended in 200 mM ammonium acetate (pH 6.9) to a final concentration of 50 μM. For native ESIBD, it was desalted by passing through two P6 buffer exchange columns (7326222, Bio-Rad), equilibrated with 200 mM ammonium acetate (pH 6.9) and diluted in 200 mM ammonium acetate (pH 6.9) to reach the concentration of 10 μM, and used without further purification.

For the preparation of plunge-frozen cryo-EM samples, it was desalted, purified, and transferred into a buffer (25 mM tris, 50 mM NaCl, 10 mM EDTA, and 2 mM MgCl_2_) using a Superdex 200 Increase 10/300 column (28990944, Cytiva Life Sciences). Ammonium acetate (7.5 M; A2706-100ML) for native MS and buffer components for the plunge-freezing solution, tris (93362-250G), NaCl (S3014-500G), EDTA (DS-100G), and MgCl_2_ (63068-250G) were also purchased from Sigma-Aldrich. All concentrations were calculated with respect to the most abundant oligomers.

### Native MS

Native MS was performed as previously described (*32*).

### Preparation of the control grid

The control sample was prepared using a grid with mesh size 200, R2/1 holey carbon without amorphous carbon from Quantifoil. Three microliters of a 2.5 μM solution was applied to the grid, followed by blotting and plunging into liquid ethane, using a manual plunger.

### Preparation of the native ESIBD grid

Grids were prepared and transferred as described in the main text. Figure S1 shows an overview of the deposition instrument described in more detail elsewhere (*32*, *37*). Figure S2 shows the cryodeposition stage and Aquilos transfer system including transfer rod, load lock, and preparation pot. At the beginning of a deposition experiment, clipped TEM grids are loaded into the shuttle at room temperature. Here, copper TEM grids from Quantifoil (C2-C15nCu40-01) with mesh size 400, R2/1 holey carbon covered with 2-nm amorphous carbon were used. The shuttle is loaded into the stage a few minutes before deposition to allow for thermalization to the stage, which was held at 130 K using a liquid nitrogen heat exchanger. While inserting the shuttle, a spring-loaded mechanism exposes the grids for deposition and makes electrical contact to control the electric potential and monitor ion current on the grids. The stage geometry prevents a direct line of sight between grids and warm surfaces to minimize contamination and radiative heating. The transfer rod is retracted to prevent heat transfer to the shuttle and to close the load-lock valve to obtain a cleaner vacuum. The pressure in the deposition chamber is 10^−9^ mbar; however, the pressure within the stage will be substantially lower, because it acts as a cryopump.

The beam energy is measured using a retarding-grid energy detector. The deposited charge is monitored by a picoammeter to control protein coverage. Protein ions are deposited with a deposition energy of 2 eV per charge, by applying a corresponding potential to the TEM grids. Close to monolayer coverage in the grid center is typically achieved in 30 min, and lower densities for efficient data collection can be found further from the center if needed. The second sample can be prepared using the same conditions or after varying the analyte, *m/z* window, or deposition energy. After completion, transfer into liquid nitrogen is done in less than 2 min as described in fig. S2.

### Movie acquisition and processing

All micrographs were collected using a Thermo Scientific Krios 300 kV cryo-TEM equipped with a BioQuantum energy filter operated at a slit width of 20 eV and a K3 direct electron detector (both Gatan), located at the COSMIC Cryo-EM facility at South Parks Road, University of Oxford, UK. Automated data acquisition was controlled using EPU software (Thermo Scientific). All movies were recorded in the tif format, using a range of defocus settings between −1 and −2.5 μm, an exposure of 34 *e*^−^/Å^2^, and a magnification of 105,000 corresponding to a pixel size of 0.83 Å.

Data were processed using cryoSPARC (*40*). After running Patch Motion Corr. and Patch CTF jobs, particles were picked using template picking, based on templates from manual picking, and extracted in 416 pixel × 416 pixel boxes scaled down by a factor of 2. After multiple rounds of 2D and 3D classification, particles were reextracted without down-scaling and final maps were produced using nonuniform refinement, based on ab initio initial volumes generated from our data. This was followed by local resolution estimation and local filtering. D2 symmetry was imposed in all steps, but using C1 symmetry resulted in only slightly lower resolution. Figures and movies of the resulting 3D EM density maps were generated using ChimeraX (*57*). Further movie acquisition and processing details for the two samples compared in the main text are specified in figs. S3 and S4 and table S1.

### Model building

A previously published model of β-galactosidase (PDB 6CVM) was used as a starting model. Fitting into the cryo-EM map obtained by conventional plunge-freezing reveals good agreement with this model. Fitting into the ESIBD cryo-EM map, however, revealed substantial differences between this model and the ESIBD cryo-EM map. To obtain a starting model for model building, non-ionic ligands and water molecules within the 6CVM model were removed, and the model was relaxed into the ESIBD map using ISOLDE, resulting in good agreement of secondary structureelements.

To obtain an atomic model of the resolved parts of β-galactosidase, the relaxed model was first manually inspected and trimmed by removing parts that had poor agreement with the density and/or consistently showed no obvious side-chain densities. The resulting trimmed model was inspected in Coot, where segments that did not fit their density were deleted and manually rebuilt using large aliphatic side chains as anchors. The model was then subjected to multiple rounds of real-space refinement in Phenix and manual building. Some residues, often near disordered regions, had poor side-chain densities and resulted in rotamer outliers. The statistics and model presented are the result of real-space refinement in Phenix.

### Computational details

MD simulations were conducted with the GROMACS suite (version 2021.6) (*58*), with the OPLS/AA forcefield and TIP4P water. The starting structure was prepared from the 6CVM PDB entry. For the dehydration simulations, the protein was prepared such that surface residues were protonated, while buried residues were set to a protonation state at neutral pH. To this end, ionizable surface residues were identified using PropKa (*59*), ordered according to their p*K*_a_ (where *K*_a_ is the acid dissociation constant) values, and protonated until an overall charge state of +44 e (according to the average experimental charge) was reached. The protein was solvated with a 2-nm hydration shell, approximately 19,500 water molecules, and centered in a cubic periodic box with an edge length of 50 nm. After steepest energy minimization, the system underwent 63 simulation windows of 500 ps. Before each simulation window, all molecules and ions above a threshold distance of 2 nm from the molecule were removed, and velocities were reinitialized at 370 K. The leap-frog integrator with a time step of 1 fs was used. Temperature was maintained constant at 370 K using the Nosé-Hoover thermostat with separate temperature coupling of protein and nonprotein groups with a coupling constant of 0.5 ps. Bond lengths to H atoms were constrained using the LINCS algorithm. Long-range Coulomb interactions were calculated using the particle mesh Ewald method, thus applying a neutralizing background charge.

For all simulations in bulk water, the protein was centered in a dodecahedral box with a 1-nm distance from the box edge. The protein was solvated with TIP4P water, and the entire simulation cell containing protein and solvent was neutralized using 44 Cl^−^ ions. After energy minimization with the steepest descent method, the system was equilibrated at 300 K for 100 ps using the velocity rescale thermostat with a time constant of 0.1 ps. Subsequently, the system was pressure-equilibrated with the Parrinello-Rahman isotropic barostat with a time constant of 2 ps. Production runs under NPT (constant number of atoms, pressure, and temperature) conditions were run for 200 ns with a time step of 2 fs.

Representative structures were identified using the GROMACS clustering method gromos with a 0.15-nm RMSD cutoff. The centroids of clustered structures in the last 50 ns (solvation and rehydration) and 10 ns (dehydration) were chosen as the representative structures shown in Fig. 4. Furthermore, the number of H bonds over the trajectory was calculated using GROMACS and RoG was calculated with the Python package MDTraj (version 1.9.7).
