# Supplementary material for: Cryo-EM of soft-landed β-galactosidase: Gas-phase and native structures are remarkably similar
Source: Sci Adv. 2024 Feb 14;10(7):eadl4628. doi: 10.1126/sciadv.adl4628 (PMC10866560; doi:10.1126/sciadv.adl4628)
Supplement: Supplementary file 1 — Figs. S1 to S9 Tables S1 and S2 Legends for movies S1 to S3 [file sciadv.adl4628_sm.pdf]

Science Advances
AAAS

Supplementary Materials for

Cryo-EM of soft-landed β-galactosidase: Gas-phase and native structures are remarkably similar

Tim K. Esser *et al.*

Corresponding author: Stephan Rauschenbach, stephan.rauschenbach@chem.ox.ac.uk

Sci. Adv. **10**, eadl4628 (2024)
DOI: 10.1126/sciadv.adl4628

The PDF file includes:

Figs. S1 to S9
Tables S1 and S2
Legends for movies S1 to S3

Other Supplementary Material for this manuscript includes the following:

Movies S1 to S3

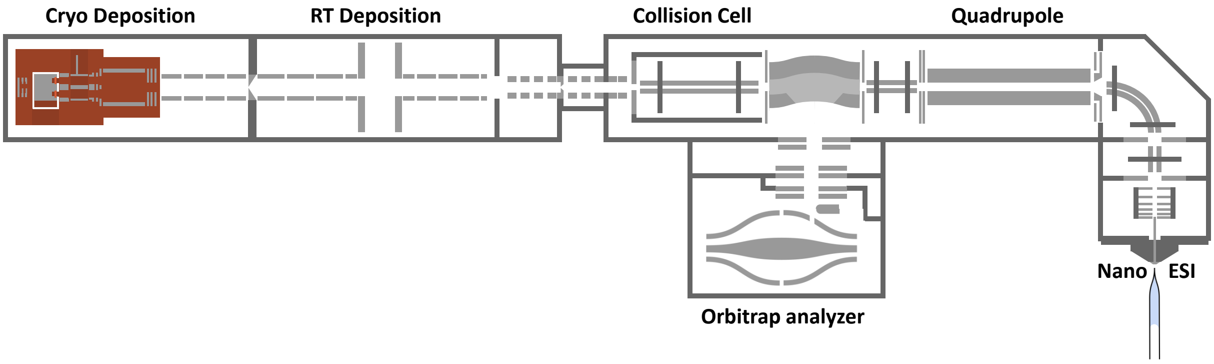

Fig. S1: **Overview of the electrospray ion-beam deposition (ESIBD) instrument.** The ESIBD instrument consists of a commercial mass spectrometer (Thermo Scientific Q Exactive UHMR instrument, right) and a custom deposition setup (left). Proteins are transferred into the gas phase using a nano electrospray ionization source. They are then mass selected using a quadrupole mass filter and a mass spectrum is recorded using the Orbitrap^{TM} detector. For cryo-EM sample preparation, the ion beam is thermalised in the collision cell and guided using electrostatic ion optics though a differential pumping chamber into the cryo deposition stage (see Fig. S2a).

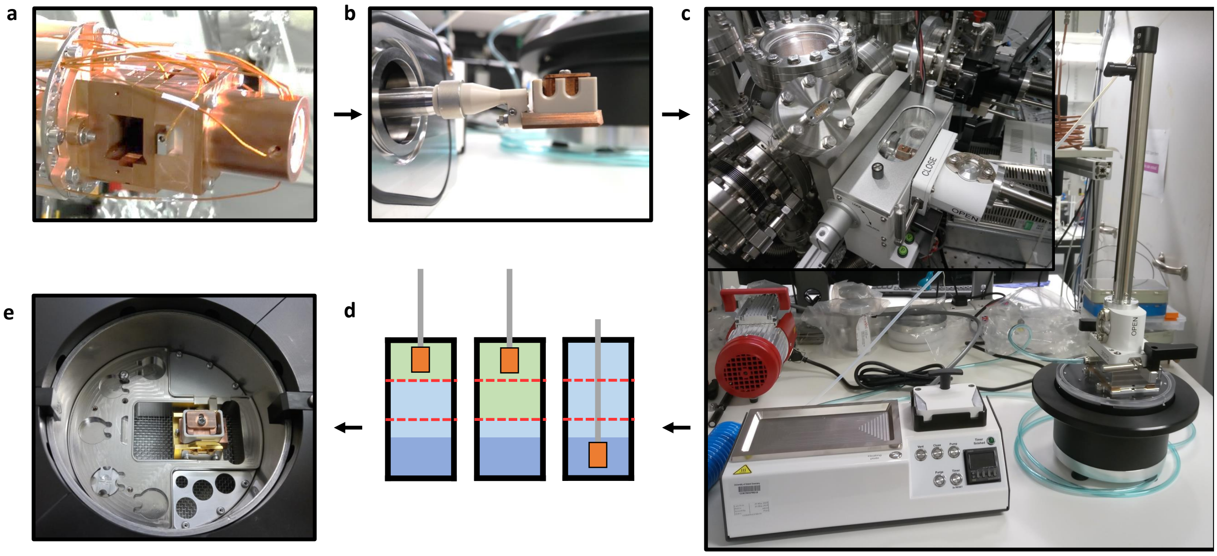

Fig. S2: **Overview of cold and clean transfer using Aquilos components. a** Picture of the cryo deposition stage. **b** Picture of the cryo shuttle. Grids are shielded by the PEEK housing during transfer. **c** Picture of sample transfer rod attached to preparation pot. The lower left shows the preparation station, used to control pumping, venting, and keeping grid handling equipment dry. The insert shows the load lock attached to the deposition chamber. **d** Schematic of cold sample transfer. Left: Cold shuttle (orange) in vacuum (light green) of transfer rod (gray). Preparation pot and load lock are purged with clean nitrogen gas (light blue). Middle: Evacuating load lock. Right: Load lock and transfer rod are vented with clean nitrogen gas. Shuttle is plunged into liquid nitrogen (blue). **e** Picture of shuttle inside preparation pot. Grids are transferred from shuttle to conventional storage boxes under liquid nitrogen (not shown.)

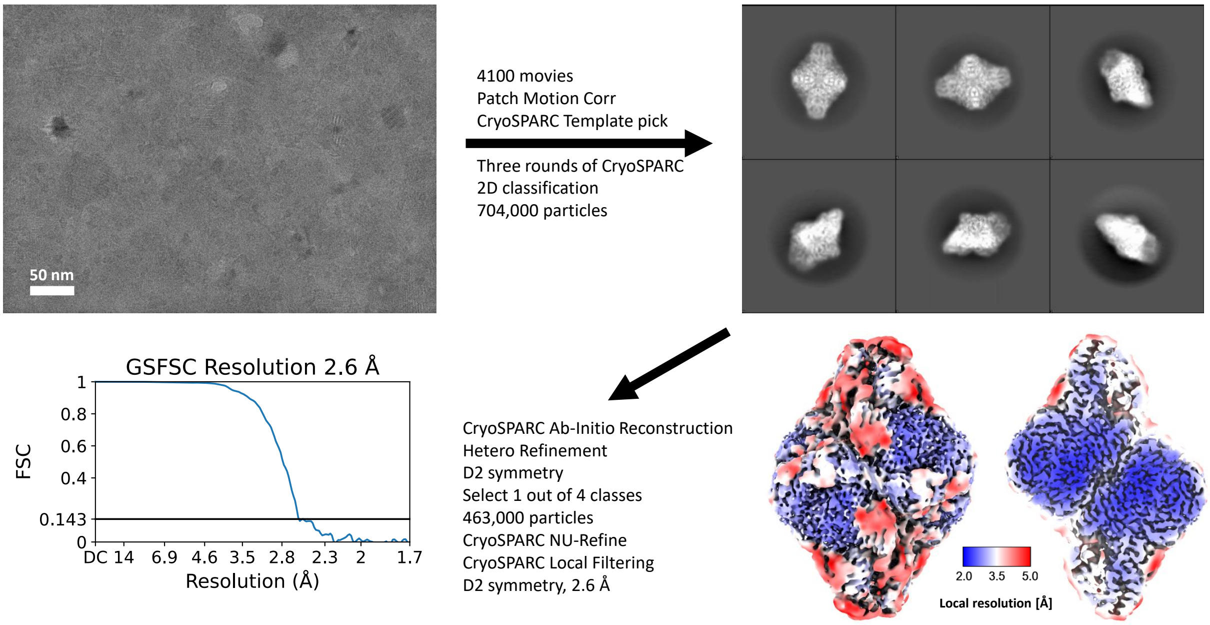

Fig. S3: **Data processing schematic for β-galactosidase from ice-coated native ESIBD sample.**. Using homogeneous refinement in CryoSPARC resulted in a 3.4 Å map. By using non-uniform refinement in CryoSPARC the resolution could be further improved to 2.6 Å.

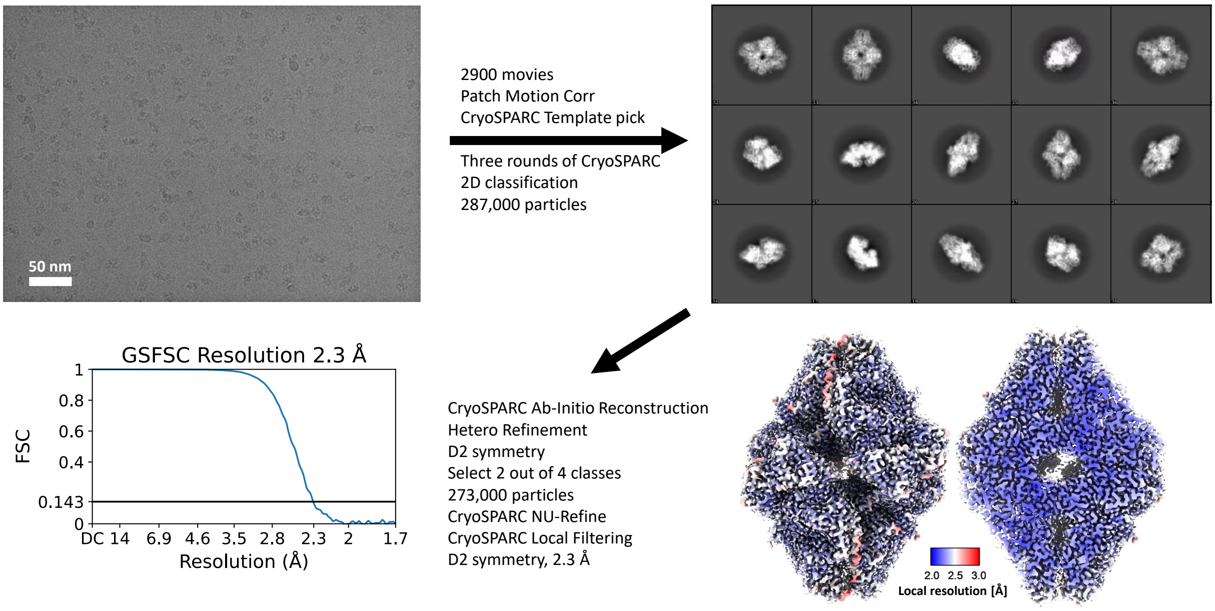

Fig. S4: **Data processing schematic for β-galactosidase from plunge-frozen control sample.**

Table S1: β-**galactosidase cryo-EM data acquisition and processing statistics.**

Data collection and processing	β-galactosidase native ESIBD	β-galactosidase solution structure
Microscope	Krios G3	Krios G3
Magnification	105,000	105,000
Voltage (kV)	300	300
Electron exposure (e^{-}/Å^2)	34	34
Defocus range (µm)	-1 to -2.5	-1 to -2.5
Pixel size (Å)	0.83	0.83
Symmetry imposed	D2	D2
Initial particle images (no.)	704,000	287,000
Final particle images (no.)	463,000	273,000
Map resolution (Å)	2.6	2.3
FSC threshold	0.143	0.143
Map resolution range (Å)	2.4 to 5.0	2.1 to 3.0
Refinement		No atomic model built
Initial model used (PDB code)	6CVM	
Model resolution (Å)	2.2/2.5/2.6	
FSC threshold	0/0.143/0.5	
Map sharpening B factor (Å^2)	-100	-84
Model composition		
Non-hydrogen atoms	10032	
Protein residues	1212	
Ligands	0	
B factors (Å^2)		
Protein	11.26	
Ligand	n/a	
R.m.s. deviations		
Bond lengths (Å)	0.003	
Bond angles (°)	0.682	
Validation		
MolProbity score	2.47	
Clashscore	13.05	
Poor rotamers (%)	3.77	
Ramachandran plot		
Favored (%)	94.62	
Allowed (%)	5.38	
Disallowed (%)	0	

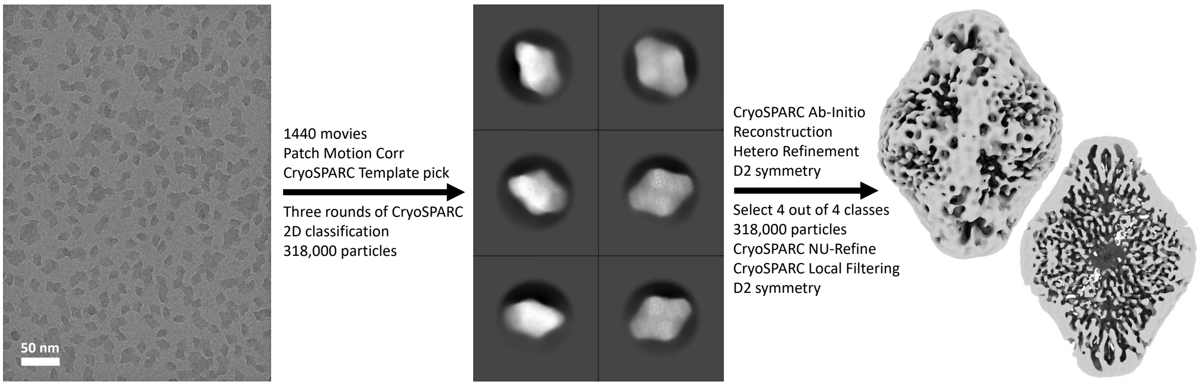

Fig. S5: **Data processing schematic for β-galactosidase from ice-free native ESIBD sample.** A map was obtained from an ice-free native ESIBD sample. The quality of this map is substantially lower than the one obtained from the ice-coated sample. In particular, high density at the edges of the map conceals the underlying protein structure. The map only shows the molecular envelope with rudimentary internal features. The outer surface of this map (including the high-density region) is substantially larger than the map obtained from the ice-coated native ESIBD sample. This may be caused by the presence of an irregular ice shell of 2 to 4 monolayers around particles. Given the pressure (10^{-7} mbar), temperature (-140 °C), and time spend in the vacuum chamber (up to 4 hours), growth of a few monolayers of ice is plausible. Alternatively, a data processing artifact caused by the high contrast of ice-free samples could be the cause. In both cases, adaptation of data analysis software might allow to obtain more information from such samples. However, we have shown that the effect can be avoided in practice by ice-coating, which provides a homogeneous background similar to conventional plunge freezing samples.

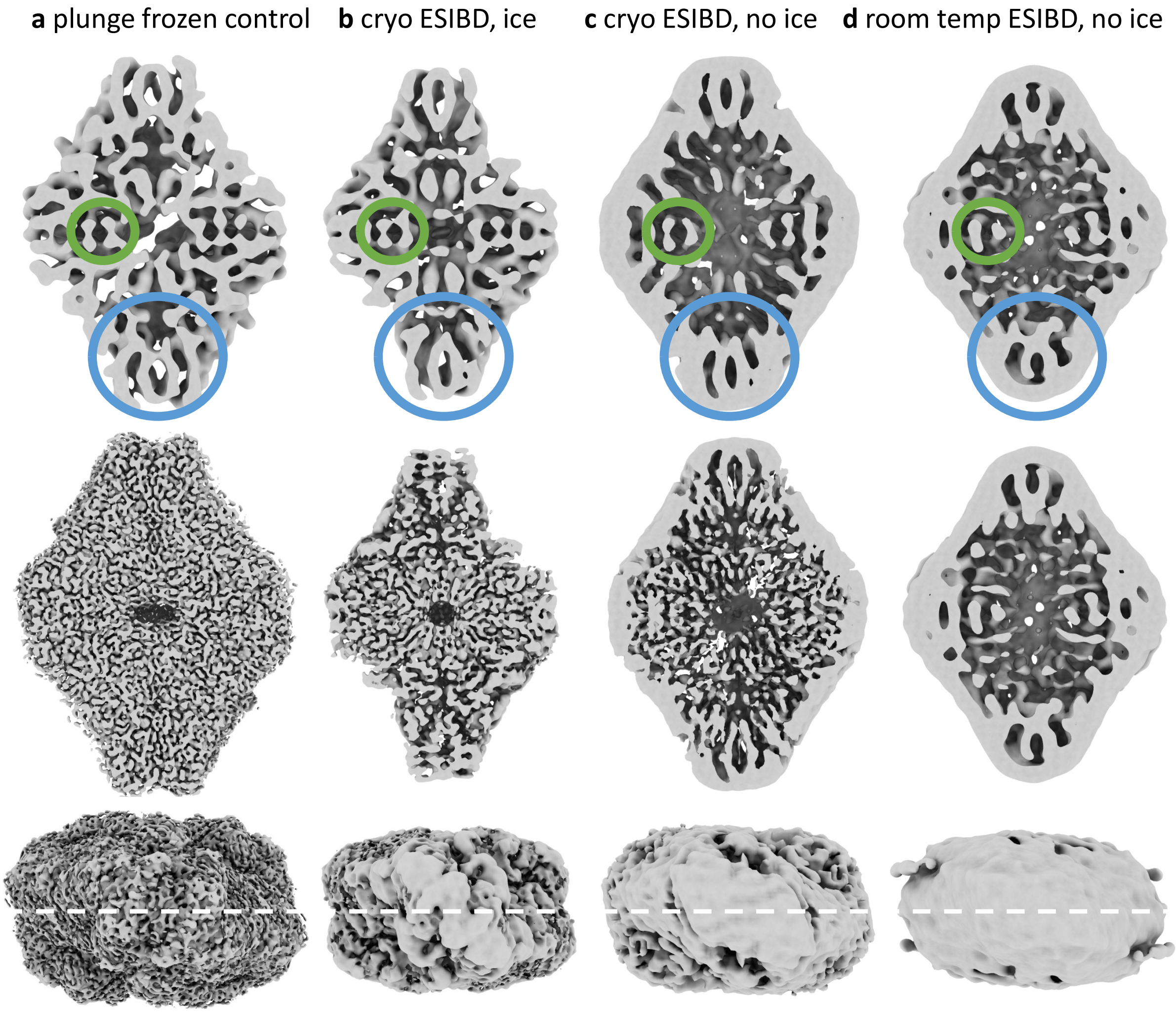

a plunge frozen control
b cryo ESIBD, ice
c cryo ESIBD, no ice
d room temp ESIBD, no ice

Fig. S6: **(Previous page) Comparison of maps for control, ice-free, ice-coated, and ice-free room-temperature deposition samples.** Maps in panels **a** and **b** are control and ice-coated native ESIBD sample, already shown in Fig. 2 in the main text. **c** Map of the ice-free native ESIBD sample from Fig. S5. **d** Map of an ice-free native ESIBD sample where deposition was done at room temperature, followed by manual transfer through ambient conditions and plunging into liquid nitrogen. This map uses data from our previous work (32), but with processing in cryoSPARC analogous to the other shown maps. The first row shows 8 Å low-pass filtered maps while the second row shows corresponding full resolution maps. The location of four characteristic central α-helices and four peripheral β-sheets are marked with green and blue circles in all 8 Å maps. The third row shows corresponding top views with a white dashed line indicating the section plane used for the sections above. While the 8 Å maps of the control and ice-coated ESIBD sample differ in overall size and size of cavities, they show similar secondary structure features as discussed in the main text. In contrast, the 8 Å maps of both ice-free samples only show rudimentary secondary structure features and exhibit the high-density shell discussed in Fig. S5, despite the differences in sample preparation workflow. Looking at the corresponding full resolution maps, the map from the sample prepared by landing on a cryogenically cooled grid without ice-coating contains more detail, though it is not sufficiently resolved to be used for model building. While landing on cryogenically cooled surfaces allowed us to obtain the 2.6 Å native ESIBD map, these observations emphasize that temperature is only one of many parameters that need to be carefully controlled to further improve the quality of native ESIBD samples.

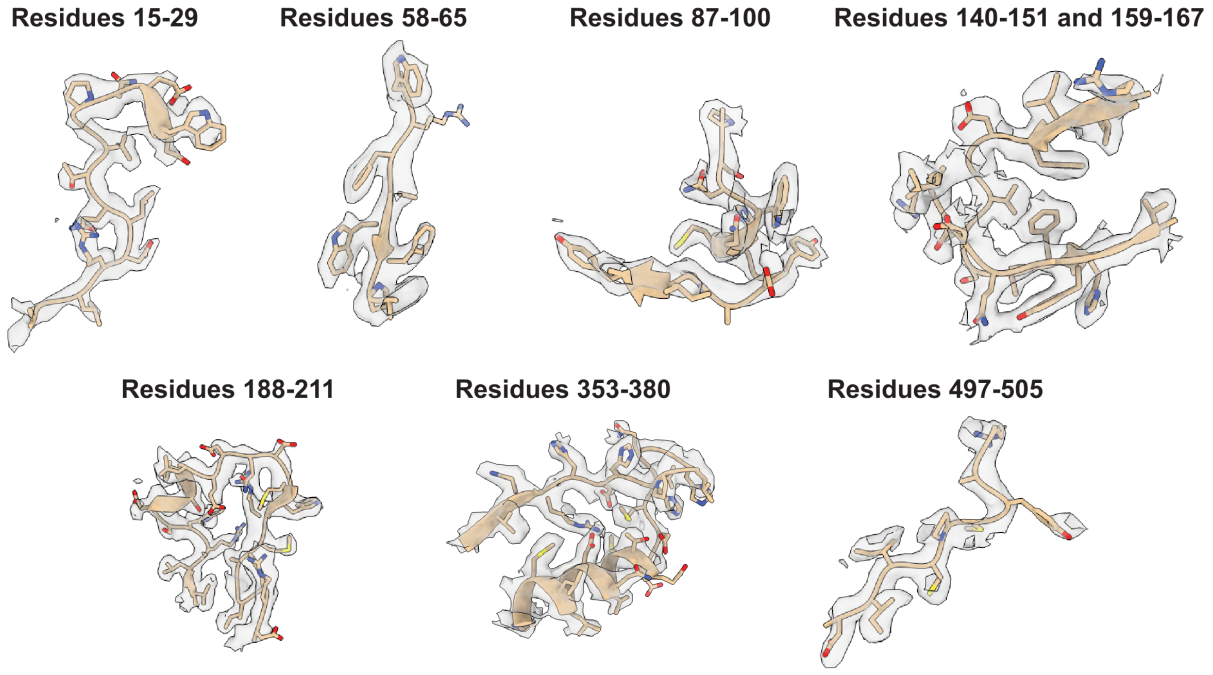

Fig. S7: **Fit of atomic model into native ESIBD map.** Gallery of segments of the manually built model are shown within the cryo-EM density, showing resolution of side chains within the map.

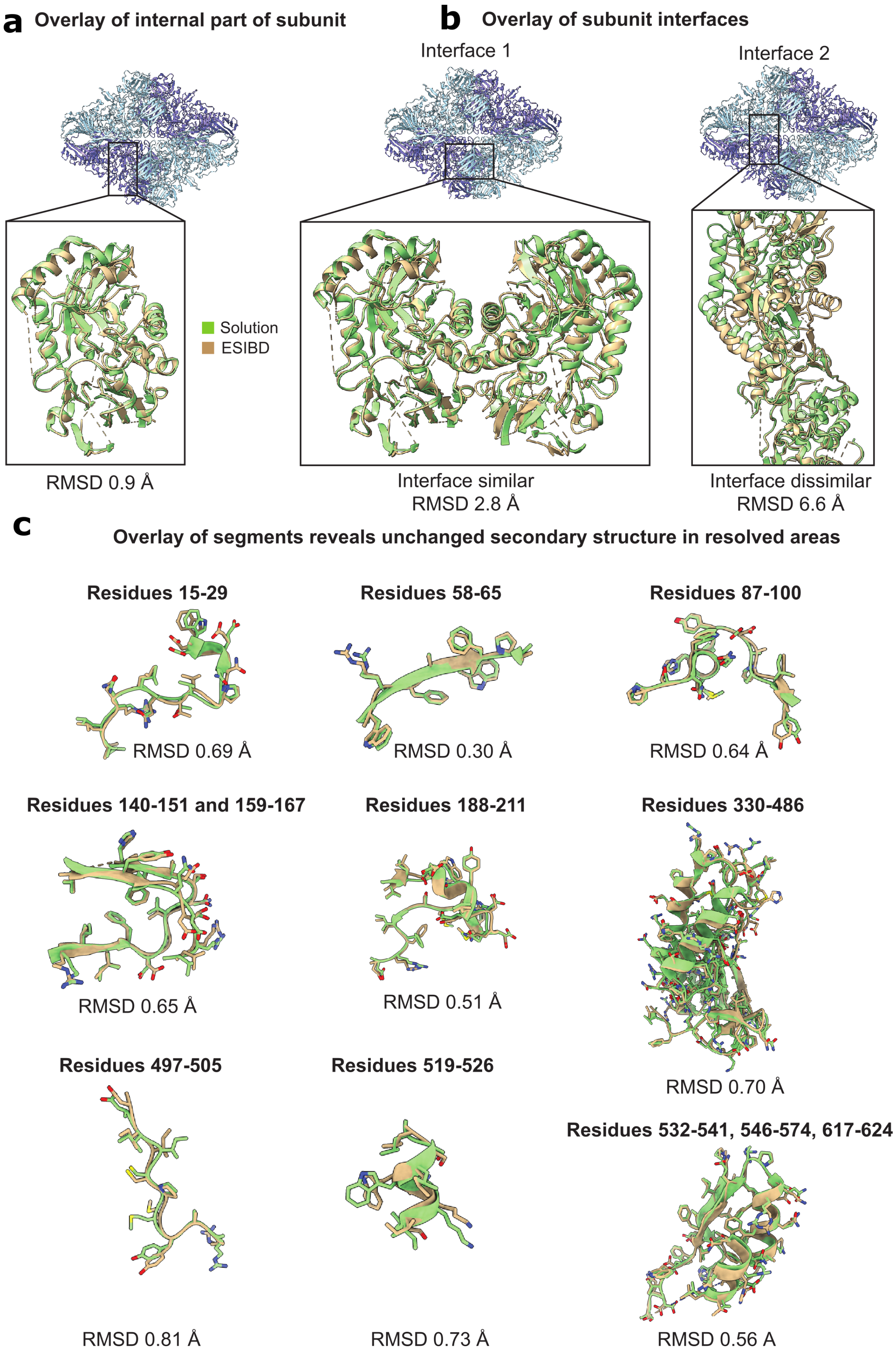
a Overlay of internal part of subunit
b Overlay of subunit interfaces
Interface 1
Interface 2
Solution
ESIBD
RMSD 0.9 Å
Interface similar
RMSD 2.8 Å
Interface dissimilar
RMSD 6.6 Å
c
Overlay of segments reveals unchanged secondary structure in resolved areas
Residues 15-29
RMSD 0.69 Å
Residues 58-65
RMSD 0.30 Å
Residues 87-100
RMSD 0.64 Å
Residues 140-151 and 159-167
RMSD 0.65 Å
Residues 188-211
RMSD 0.51 Å
Residues 330-486
RMSD 0.70 Å
Residues 497-505
RMSD 0.81 Å
Residues 519-526
RMSD 0.73 Å
Residues 532-541, 546-574, 617-624
RMSD 0.56 A

Fig. S8: **(Previous page) Comparison of built segments of native ESIBD β-galactosidase (tan) versus solution structure (green).** **a** Comparison of a monomeric unit core region, aligned using the *matchmaker* function in ChimeraX. RMSD = 0.9 Å. **b** Left: Changes in the first type of interface between subunits in β-galactosidase. RMSD = 2.8 Å. Right: Changes in the second type of interface between subunits in β-galactosidase. RMSD = 6.6 Å. **c** Agreement between segments within one monomeric subunit, demonstrating conserved secondary structure. Segments were aligned and root mean square deviation was calculated using the *matchmaker* function in ChimeraX.

Table S2: **Deviation between MD and experimental structures.** The table provides Cα RMSD values between structures shown in Fig. 4 in the main text, as obtained using the align command in ChimeraX. The deviations are in line with the changes in radius of gyration and number of hydrogen bonds shown in Fig. 4. However, the global RMSD does not capture the local similarities and deviations shown in Fig. S7 and Fig. S8.

Compared structures	RMSD (Å)
Solvated MD vs. 6CVM (PDB)	3.25
Dehydrated MD vs 6CVM (relaxed to ESIBD map)	5.16
Rehydrated MD vs. 6CVM (PDB)	4.62
Solvated MD vs. Dehydrated MD	7.18
Solvated MD vs. Rehydrated MD	5.31
Dehydrated MD vs. 6CVM (PDB)	5.78
6CVM (PDB) vs. 6CVM (relaxed to ESIBD map)	7.55

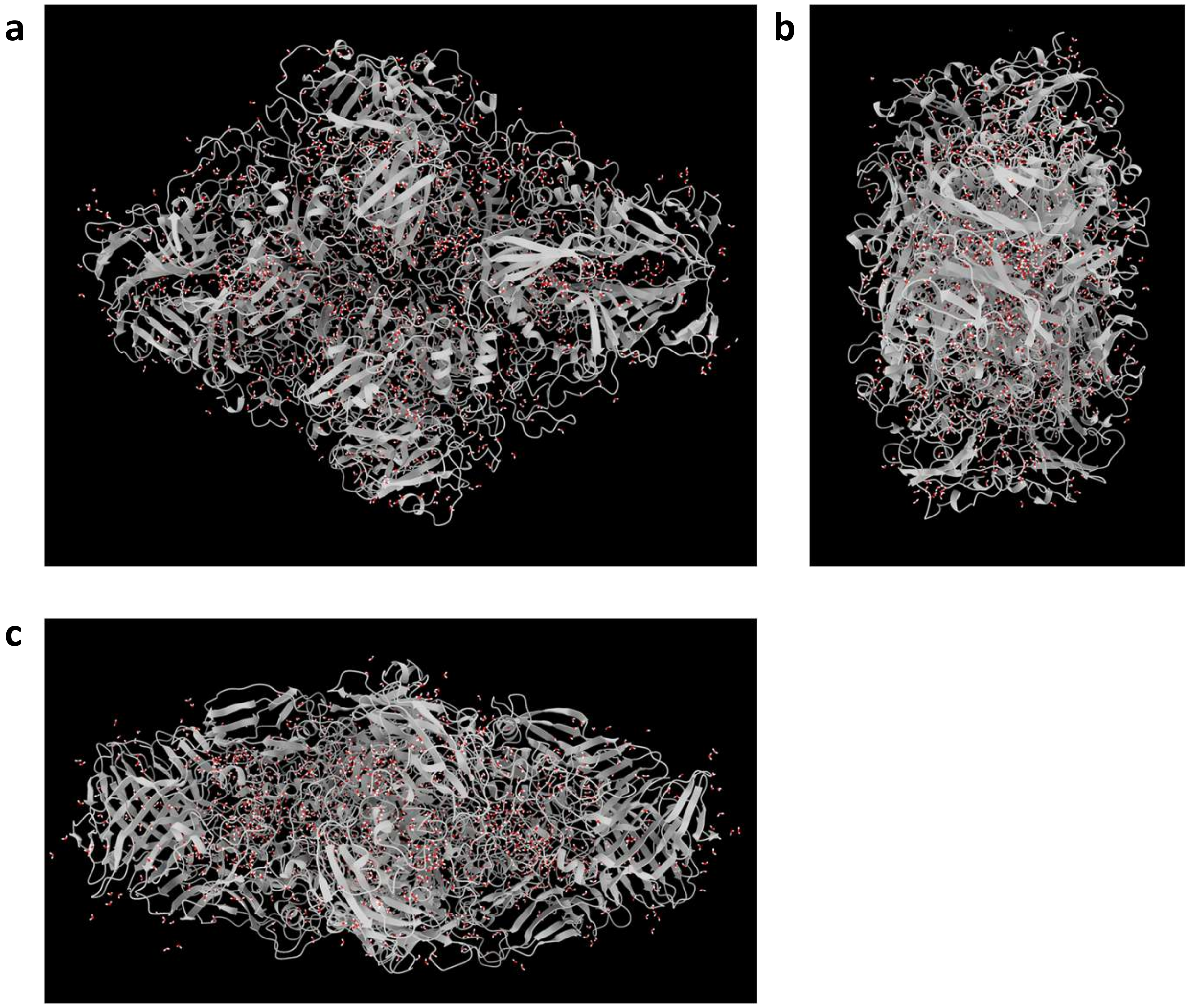

Fig. S9: **Position of water molecules in dehydrated MD structure.** Panels **a**, **b**, and **c** show the dehydrated MD structure from the three principal directions. Most remaining 1,400 water molecules are found inside the protein structure. We have previously estimated the number of water molecules remaining in the experiment to less than 200 (32). These 1,400 remaining water molecules are substantially less than the combined 4,194 structural water molecules found in the solvated structure (PDB 6CVM) and 4,200 molecules comprising a 3 Å solvation shell that could preserve the solvated structure (20). The different degrees of solvation in the dehydrated MD and native ESIBD structures can account for some of the structural differences between them. However, they both clearly represent substantially dehydrated states with respect to the native solvated structure.

Movie 1: Animation of the 2.6 Å native ESIBD map.

Movie 2: Animation of maps from native ESIBD (2.6 Å) and plunge-frozen control (2.3 Å), corresponding models and morph between models.

Movie 3: Morph between initial, dehydrated, and rehydrated MD models compared to corresponding experiment based solvated and relaxed PDB models.
